# Method validation studies and an inter-laboratory cross validation study of lenvatinib assay in human plasma using LC-MS/MS

**DOI:** 10.1016/j.plabm.2018.e00103

**Published:** 2018-05-24

**Authors:** Yuji Mano

**Affiliations:** Drug Metabolism and Pharmacokinetics, Eisai Co., Ltd., Tokodai 5-1-3, Tsukuba-shi, Ibaraki 300-2635, Japan

**Keywords:** Validation, Cross validation, Lenvatinib, LC-MS, Human plasma

## Abstract

Cross validation studies for bioanalytical methods are important to ensure that assay data from all study sites where sample analysis is performed can be compared throughout clinical trials. To support global clinical studies of lenvatinib, a novel multi-targeted tyrosine kinase inhibitor, seven bioanalytical methods by liquid chromatography with tandem mass spectrometry (LC-MS/MS) were developed at five laboratories. In this study, methods were initially validated at each laboratory according to bioanalytical guidelines. For subsequent inter-laboratory cross validation, quality control (QC) samples and clinical study samples with blinded lenvatinib concentrations were assayed to confirm comparable assay data. Lenvatinib and an internal standard were extracted by protein precipitation, liquid-liquid extraction, or solid phase extraction and then detected in positive ion electrospray mode by multiple reaction monitoring using LC-MS/MS. The assay method developed at each laboratory was successfully validated with parameters within the acceptance criteria recommended by the guidelines. In the cross validation study, accuracy of QC samples was within± 15.3% and percentage bias for clinical study samples was within± 11.6%. These findings suggest that lenvatinib concentrations in human plasma can be compared across laboratories and clinical studies.

## 1 Introduction

Lenvatinib is a novel multi-targeted tyrosine kinase inhibitor which inhibits vascular endothelial growth factor receptors 1–3, fibroblast growth factor receptors 1–4, platelet-derived growth factor receptor α, c-Kit, and RET (rearranged during transfection) [1], [2], [3]. Lenvatinib is registered as LENVIMA^®^ to treat patients with progressive, differentiated thyroid cancer as monotherapy and advanced renal cell carcinoma in as combination therapy with everolimus. Prior to regulatory approval, a number of clinical studies were conducted across the globe and five bioanalytical laboratories established assay methods for the determination of lenvatinib concentrations in human plasma. To the best of our knowledge, two bioanalytical methods for lenvatinib have been reported; lenvatinib and its metabolites assay in human plasma, urine and faeces [4] and lenvatinib assay in human serum and phosphate buffered saline for protein binding studies [5].

In this study, bioanalytical methods for the determination of lenvatinib concentrations in human plasma are reported. The first objective of this study is to show a comprehensive list of assay methods for lenvatinib and its validation parameters at each laboratory. The second objective of this study is to assess whether all the bioanalytical laboratories of lenvatinib assay demonstrate comparable data to ensure that pharmacokinetic parameters of lenvatinib can be compared across clinical trials. Although successful validation of each method is a pre-requisite to performing clinical sample assay, cross validation studies are also required to compare concentrations among bioanalytical methods and/or laboratories as per the bioanalytical guidelines by European Medicines Agency [6] and U.S. Food and Drug Administration [7]. In addition to satisfying regulatory requirement, the scientific rationale for performing cross validation studies is to ensure that bioanalytical data are comparable among laboratories when multiple bioanalytical methods are used and pharmacokinetics are compared across clinical trials. Cross validation has been done in many reported studies in which clinical study samples (post-dose samples) with unknown concentrations [8], [9], [10], [11], [12], [13] or quality control (QC) samples with known concentrations [14], [15], or both [16], [17] were quantified. In the present inter-laboratory cross validation study, QC samples prepared by a central laboratory were assayed using seven validated methods at five bioanalytical laboratories. In addition, clinical study samples were analyzed between two laboratories to further support data comparability.

## 2 Materials and methods

### 2.1 Materials

**Fig. 1:**
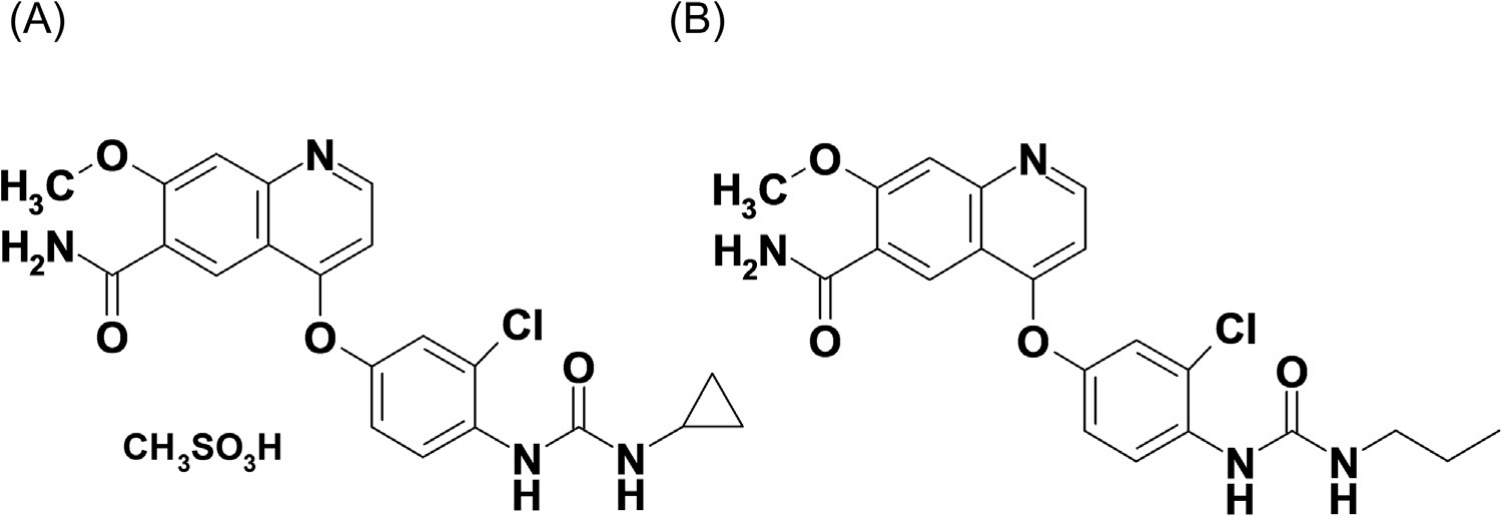
Chemical structures of lenvatinib (A) and ER-227326, an intern al standard, (B).

Lenvatinib and ER-227326 (Fig. 1), a structural analogue internal standard (IS), were synthesized at Eisai Co., Ltd (Ibaraki, Japan), while a ^13^C_6_ stable isotope labeled lenvatinib used as an IS was synthesized at Wuxi AppTec (Shanghai, China). Drug-free blank human plasma with heparin sodium as an anticoagulant, purchased from commercial vendors or obtained in-house with written consent, was used to prepare calibration standards and QC samples. Commercially available analytical grade, special grade, or high-performance liquid chromatography grade reagents were used.

### 2.2 Preparation of calibration and quality control samples

Lenvatinib was dissolved in methanol/water (1/1, v/v) in the methods A, C, E1, E2, and E3, or methanol in methods B and D to prepare stock solutions which were then serially diluted to make working solutions (Table 1). Working solutions of lenvatinib at equal to or more than seven concentration levels covering the quantifiable range were prepared at each laboratory. The stock and working solutions prepared by methanol were stored at − 20 or − 70 °C while those prepared by methanol/water (1/1, v/v) were stored refrigerated. The solutions were used within the period ensured in the method validation studies. Calibration samples were prepared by spiking an aliquot of the working solution into blank human plasma at each laboratory. In the method validation studies at each laboratory, QC samples including the lower limit of quantification (LLOQ), low QC (LQC), mid QC (MQC), high QC (HQC), or the upper limit of quantification (ULOQ) samples were prepared. In cross validation studies, routine QC samples at three or four concentrations, including LQC, MQC, and HQC were prepared at each laboratory to ensure assay run acceptance. The calibration range and concentrations of QC samples are presented in Table 1.

**Table 1:** Summary of lenvatinib assay methods in human plasma.

**Laboratory**	**A**	**B**	**C**	**D**	**E**		
**Region**	Asia	US	US	EU	Asia		
**Method**	A	B	C	D	E1	E2	E3
**Assay sample volume (mL)**	0.2	0.05	0.1	0.2	0.1	0.1	0.1
**Assay range (ng/mL), number of calibrators**	0.1–500, 9 points	0.25–250, 8 points	0.25–250, 7 points	0.1–100, 8 points	0.25–500, 7 points	0.25–250, 7 points	0.25–250, 7 points
**IS**	ER-227326	^13^C_6_ lenvatinib	^13^C_6_ lenvatinib	ER-227326	ER-227326	^13^C_6_ lenvatinib	ER-227326
**Standard solution**	50% methanol	Methanol	50% methanol	Methanol	50% methanol	50% methanol	50% methanol
**Sample extraction and its volume**	LLE by diethyl ether, 2.5 mL	PP by ACN-MeOH (2:1), 0.3 mL	LLE by MTBE-IPA(7:3) with 0.1% AA, 0.75 mL	LLE by diethyl ether, 1.5 mL	SPE by HLB plate (10 mg), 0.4 mL of 0.2 mM ammonium acetate in ACN with 1%FA	LLE by MTBE-IPA (7:3) with 0.1% AA, 0.75 mL	SPE by MCX plate (10 mg), 0.4 mL of 5% NH_4_OH in ACN/Water (75:25, v/v)
**Reconstitution solvents and its volume**	MeOH-water (1:1, v/v), 1.0 mL	FA-MeOH-water (1:200:800, v/v/v), 0.2 mL	FA-ACN (1:1000, v/v): FA-water (1:1000, v/v)= 1:9, 0.2 mL	MeOH-water (1:1, v/v), 0.5 mL	AA-MeOH-water (1:150:850, v/v/v), 0.2 mL	FA-ACN (1:1000, v/v): FA-water (1:1000, v/v)= 1:9, 0.2 mL	(A) 0.5% FA (B) ACN-MeOH with 0.5% FA (25:75, v/v) (A:B, 7:3, v/v), 0.2 mL
**Injection volume (μL)**	5	10	10	20	7	5	5
**Chromatography**	RP-HPLC (Shimadzu), Column: Symmetry Shield RP8 column (2.1 × 150 mm, 3.5 µm), Mobile phase: 2 mM NH_4_Ac (pH 4.0)-ACN (3:2)	RP-HPLC (Shimadzu), Column: Hypersil Gold column (2.1 × 50 mm, 5 µm), Mobile phase: (A) 0.1% FA (B) MeOH with 0.1% FA	RP-HPLC (Shimadzu), Column: Synergi Polar-RP column (2.0 × 50 mm, 4 µm), Mobile phase: (A) 0.2% FA (B) ACN/MeOH with 0.2% FA	RP-HPLC (Agilent), Column: Symmetry Shield RP8 column (2.1 × 150 mm, 3.5 µm), Mobile phase: (A) 40%ACN with 2 mM NH_4_Ac (pH 4.0) (B) ACN	RP-HPLC (Agilent), Column: Synergi Polar-RP column (2.0 × 50 mm, 4 µm), Mobile phase: (A) 0.2 mM NH_4_Ac with 0.1% AA (B) MeOH-IPA (19:1) with 0.1% AA and 0.2 mM NH_4_Ac	RP-HPLC (Agilent), Column: Synergi Polar-RP column (2.0 × 50 mm, 4 µm), Mobile phase: (A) 0.2% FA (B) ACN-MeOH (9:1) with 0.2% FA	RP-HPLC (Agilent), Column: Luna C18(2) column (2.0 × 50 mm, 5 µm), Mobile phase: (A) 0.5% FA, (B) ACN-MeOH (25:75) with 0.5% FA
**Elution**	Flow rate: 0.2 mL/min	Flow rate: 0.4 mL/min	Flow rate: 0.35 mL/min	Flow rate: 0.2 mL/min	Flow rate: 0.3 mL/min	Flow rate: 0.35 mL/min	Flow rate: 0.45 mL/min
	Isocratic elution	Gradient elution; Mobile phase B (%):	Gradient elution; Mobile phase B (%):	Gradient elution; Mobile phase B (%):	Gradient elution; Mobile phase B (%):	Gradient elution; Mobile phase B (%):	Gradient elution; Mobile phase B (%):
		35% (0 min),	10% (0 min),	0% (0 min),	15% (0 min),	10% (0 min),	20% (0 min),
		35% (0.5 min),	10% (0.2 min),	100% (6.5 min), 0% (7.25 min), 0% (12.0 min)	15% (0.75 min),	10% (0.2 min),	20% (0.01 min),
		95% (1.5 min),	90% (0.75 min), 100% (1.5 min), 100% (2.6 min), 10% (2.61 min), 10% (4.0 min)		65% (1.75 min),	90% (0.75 min),	70% (1.8 min),
		95% (3.0 min),			65% (4.0 min),	100% (1.5 min),	100% (1.81 min),
		35% (3.1 min),			100% (4.2 min),	100% (2.6 min),	100% (2.4 min),
		35% (3.5 min),			100% (5.4 min),	10% (2.61 min),	20% (2.41 min),
		35% (4.5 min)			15% (5.5 min),	10% (4.0 min)	20% (3.7 min)
					15% (7.0 min)		
**MS Detection**	API4000, MRM positive, precursor/product ions *m/z* 427/370 (LEN), 429/370 (IS)	API4000, MRM positive, precursor/product ions *m/z* 427.1/370.0 (LEN), 433/376 (IS)	API4000, MRM positive, precursor/product ions *m/z* 427/370 (LEN), 433/376 (IS)	API3000, MRM positive, precursor/product ions *m/z* 427/370 (LEN), 429/370 (IS)	API5000, MRM positive, precursor/product ions *m/z* 427.1/370.1 (LEN), 431.1/372.1 (IS)	API4000, MRM positive, precursor/product ions *m/z* 427.1/370.0 (LEN), 433/376 (IS)	API5000, MRM positive, precursor/product ions *m/z* 427.1/370.1 (LEN), 431.2/372.1 (IS)
**QC concentrations in cross validation (ng/mL)**	0.25, 20, 320	0.75, 15, 100, 200	0.75, 30, 190	0.25, 5, 80	0.75, 5, 375	0.75, 30, 190	0.6, 10, 200
**QC concentrations in method validation (ng/mL)**	0.1, 0.2, 20, 500	0.25, 0.75, 15, 100, 200	0.25, 0.75, 30, 190	0.1, 0.25, 5, 80	0.25, 0.75, 5, 375, 500	0.25, 0.75, 30, 190, 250	0.25, 0.6, 10, 200

AA: Acetic acid; Ac: Acetate; ACN: Acetonitrile; FA: Formic acid; IPA: Isopropanol; IS: Internal standard; LEN: Lenvatinib; LLE: liquid-liquid extraction; MeOH: Methanol; MRM: Multiple reaction monitoring; MS: Mass spectrometry; MTBE: Methyl t-butyl ether; PP: Protein precipitation; QC: Quality control; RP-HPLC: Reverse phase high-performance liquid chromatography; SPE: Solid phase extraction.

### 2.3 Sample processing and assay conditions

HPLC systems from Agilent (CA, USA) and Shimadzu (Kyoto, Japan) were used as liquid chromatography (LC) systems. Lenvatinib was quantified with a triple quadrupole mass spectrometer from Sciex (MA, USA) in the positive ion electrospray mode by multiple reaction monitoring. Details on sample processing and assay conditions of each method are summarized in Table 1.

### 2.4 Method validation

At each laboratory, an established method for the determination of lenvatinib in human plasma was validated in compliance with the bioanalytical regulatory guidelines from European Medicines Agency [6] and US Food and Drug Administration [7]. The following validation parameters were assessed: selectivity, carryover, intra- and inter-batch reproducibility, dilution integrity, extraction recovery, matrix effects, and stability. Acceptance criteria for each parameter was in accordance with those in the guidelines.

### 2.5 Cross validation

#### 2.5.1 Assay run evaluation using calibration and QC samples

Calibration curves were acquired by weighted regression plotting the peak area ratios (PARs) of lenvatinib to the IS and the corresponding nominal concentrations of calibration samples. Types of regression and weighting factors are summarized in Table 2. Linear regression was employed in all the methods, except the method E1 where quadratic regression was used. Weighting factor was 1/concentration^2^, except in the method A where 1/PAR^2^ was used. Routine QC samples at three or four concentration levels prepared at each laboratory were assayed with cross validation samples in analytical runs to ensure assay run acceptance. The percentage of relative error (% RE) of calibration samples should be within± 15% ( ± 20% was allowed for the LLOQ). % RE of routine QC samples should be within± 15%.

**Table 2:** Summary of lenvatinib assay validation parameters.

Method	A	B	C	D	E1	E2	E3
Selectivity	No interferences	No interferences	No interferences	No interferences	No interferences	No interferences	No interferences
Carryover	No carryover	No carryover	Minimal carryover	NT	> 20% of the LLOQ	> 20% of the LLOQ	No carryover
Regression and weighting	Linear regression with weighting (1/PAR^2^)	Linear regression with weighting (1/conc^2^)	Linear regression with weighting (1/conc^2^)	Linear regression with weighting (1/conc^2^)	Quadratic regression with weighting (1/conc^2^)	Linear regression with weighting (1/conc^2^)	Linear regression with weighting (1/conc^2^)
Precision in intra-batch	0.3–9.8%	1.6–8.2%	1.2–7.2%	0.8–7.3%	4.2–9.0%	1.2–14.9%	0.9–8.7%
Accuracy in intra-batch	− 11.1 to 3.8%	− 8.3 to 2.3%	− 2.1 to 0.0%	− 2.9 to 3.6%	− 4.0 to 5.4%	− 5.2% to − 0.3%	− 3.5 to 10.4%
Precision in inter-batch	2.2–11.9%	1.5–6.1%	2.1–5.9%	3.3–8.0%	4.2–10.3%	3.4–12.7%	3.5–11.8%
Accuracy in inter-batch	− 11.5 to 7.4%	− 9.0 to 1.7%	− 4.2 to 2.0%	− 3.0 to 1.5%	− 3.9 to 4.8%	− 3.3 to 1.0%	− 7.3 to 6.7%
Dilution integrity (concentrations before dilution)	Up to 100-fold (20,000 ng/mL)	Up to 50-fold (400 ng/mL)	Up to 10-fold (400 ng/mL)	Up to 10-fold (511 ng/mL)	NT	Up to 10-fold (400 ng/mL)	Up to 20-fold (500 ng/mL)
Extraction recovery	64.8–72.5%	43.4–52.7%	ca. 100%	NT	91.4–94.2%	86.2–90.1%	94.7–98.1%
Matrix effects	No matrix effects	No matrix effects	No matrix effects	NT	No matrix effects	No matrix effects	No matrix effects
Stability	Bench-top in plasma for 24 h; up to 3 F/T cycles in plasma; Frozen in plasma at − 20 °C for 322 days; processed samples at 10 °C for 72 h; stock solution at 4 °C for 35 days	Bench-top in plasma for 22 h; up to 5 F/T cycles in plasma; Frozen in plasma at − 20 °C for 209 days; processed samples at 4 °C for 123 h; stock solution at − 20 °C for 544 days	processed samples at 8 °C for 38 days	Bench-top in plasma for 24 h; up to 3 F/T cycles in plasma; Frozen in plasma at − 20 °C for 19.5 month; processed samples at 2–8 °C for 7 days; stock solution at − 70 °C for 150 days	Bench-top in plasma for 20 h; processed samples at 8 °C for 94 h; stock solution at RT for 25 h; 136 days at 2–8 °C	Bench-top in blood for 8 h; up to 5 F/T cycles in plasma; Frozen in plasma at − 20 °C for 790 days; processed samples at 8 °C for 153.5 h; stock solution at RT for 67 h; stock solution at − 20 °C for 366 days	Up to 5 F/T cycles in plasma; Processed samples at 20 °C for 94 h;

Conc: Concentration; F/T: Freeze-thaw; LLOQ: Lower limit of quantification; NT: Not tested; PAR: Peak area ratio; RT: Room temperature.

#### 2.5.2 Assay of cross validation samples

Cross validation QC samples at four lenvatinib concentrations (0, 0.5, 10, and 200 ng/mL, three replicates per concentration) were prepared at a central laboratory (Laboratory A) and shipped with concentration blinded to laboratories B, C and E after ensuring its accuracy by a validated assay method A. The QC samples for the cross validation study have been shipped from the laboratory A to other laboratories under frozen conditions (typically at −70 °C) via international courier. The samples received at each laboratory were frozen without any other issues. Lenvatinib concentrations of cross validation QC samples were determined at each laboratory using respective fully validated bioanalytical methods. Once the assay data were finalized, the nominal concentrations were informed to each laboratory for calculation of accuracy. Prior to the multi-laboratory cross validation study, a separate cross validation study between laboratories A and D was performed using both post-dose samples from a clinical study and QC samples. Cross validation QC concentrations at three concentrations (0.25, 20, and 320 ng/mL) and a total of 12 post-dose samples were assayed at two laboratories. For comparison of assay data of post-dose samples, percentage bias between the two laboratories was calculated as described in our previous report [12].

## 3 Results

### 3.1 Method validation

Validation parameters of each method are summarized in Table 2. The method validation studies at each laboratory were performed in accordance with the regulatory guidelines [6], [7].

#### 3.1.1 Selectivity and carryover

Selectivity of the methods was assessed using blank plasma from six individuals to ensure no interference peaks at the retention times of lenvatinib and the IS. No interfering peaks were detected in blank chromatograms at the eluted positions of lenvatinib and the IS. Lenvatinib peaks with sufficient signal-to-noise ratio were noted even at the LLOQ level. Carryover was evaluated by injecting blank samples just after the ULOQ samples. Carryover was noted in the methods E1 and E2 while no or minimal carryover was observed in the other methods. In those two methods, peaks with area more than 20% of that of the LLOQ sample were detected at the retention time of lenvatinib, which was out of the acceptance criteria of the bioanalytical guidelines. This finding triggered further investigation on its impacts in each sample assay.

#### 3.1.2 Intra- and inter-batch reproducibility

Five or six replicates per concentration at four or five QC levels including the LLOQ, LQC, MQC, and HQC (or ULOQ) were assessed in the intra-batch reproducibility study to assess the accuracy and precision. The inter-batch reproducibility study consisted of three separate intra-batch assessments. Precision and accuracy in the intra-assay batch were within 14.9% and± 11.1%, respectively. In the inter-batch reproducibility, precision and accuracy were not more than 12.7% and± 11.5%, respectively. These were within the acceptance criteria recommended by the guidelines (15% and± 15% for precision and accuracy, respectively, for LQC, MQC, and HQC (or ULOQ), 20% and± 20% for precision and accuracy, respectively, for the LLOQ) [6], [7].

#### 3.1.3 Dilution integrity

For assaying samples with concentrations above the ULOQ, samples should be diluted by blank plasma so that concentrations are within the validated quantifiable range. Various dilution factors were evaluated in each method: up to 10-fold in the methods C, D, and E2; up to 20-fold in the method E3; up to 50-fold in the method B; and up to 100-fold in the method A. Concentrations of QC samples before dilution have been shown in Table 2. Accuracy and precision of QC samples diluted with designated dilution factors were within± 15% and 15%, respectively, suggesting that the dilution integrity was confirmed.

#### 3.1.4 Extraction recovery and matrix effects

Extraction recovery of lenvatinib should be consistent irrespective of the concentrations tested and preferably similar to that of the IS. Regardless of the extraction methods – including protein precipitation, liquid-liquid extraction, and solid-phase extraction – the extraction recovery of lenvatinib was middle or high. The extraction recovery of lenvatinib was greater than 60% while that in the method B was relatively lower (ca. 50%). Although reasons for the lower recovery in the method B remain to be investigated, the low recovery does not impact the lenvatinib quantification since the recovery is consistent among the concentrations tested which covered clinical relevant lenvatinib levels. The extraction recoveries of the IS in the methods A, B, E1, E2, and E3 were 82.6%, 48.3%, 76.8%, 89.6%, and 90.7%, respectively, which were similar to those of lenvatinib.

Matrix effects are important validation parameters in the assay method using LC-MS/MS. Matrix factors were evaluated using human plasma from six individuals and determined by division of peak area of the analyte of interest in extracted blank fortified with neat solution of reference standard by that in neat solution with the same concentration. The CV of matrix factors in plasma from six individuals should be within 15% to ensure “no matrix effects”. The matrix factors of lenvatinib itself were 1.11 with % CV of 0.4%, 053–0.59 with % CV within 5.9%, 0.55–0.67 with % CV within 7.1%, 0.69–0.80 with % CV within 6.5%, and 1.14–1.27 with % CV within 4.1% in the methods B, C, E1, E2, and E3, respectively. In the method A, accuracy of QC samples at 0.5 ng/mL using plasma from six individuals was determined and the accuracy was within± 13.9%, indicating that matrix did not impact accuracy of lenvatinib assay. The total recovery of lenvatinib is defined by multiplying extraction recovery and matrix factors of lenvatinib. The total recovery of lenvatinib was approximately 0.54, 0.57–0.64, 0.55, 0.60–0.70, and 1.15, in the methods B, C, E1, E2, and E3, respectively, given that extraction recoveries were 0.484, 1.078, 0.926, 0.877, and 0.966, respectively. The total recovery was similar among the methods B, C, E1, and E2, while higher in the method E3. The difference in the total recovery among the five methods is attributable to differences in matrix effects given that the extraction recoveries in the methods C, E1, E2, and E3 were similar.

#### 3.1.5 Stability

Various stability parameters were assessed including bench-top stability in plasma at ambient temperature, freeze-thaw stability in plasma, frozen stability in plasma, stability in processed samples, and stability in standard solutions. The following results with the longest stability duration were obtained and were method-independent: lenvetinib in human plasma was stable up to 24 h at room temperature; freeze/thaw stability in plasma was ensured after 5 cycles; frozen stability in plasma was confirmed for 790 days at − 20 °C; lenvatinib in stock solutions in methanol and methanol/water (1/1, v/v) at − 20 °C were stable for 544 and 366 days, respectively, while in methanol/water (1/1, v/v) at 2–8 °C were stable for 136 days. The stability of lenvatinib in processed samples was method-dependent and was ensured to cover the duration of an analytical batch at each laboratory.

### 3.2 Cross validation

The calibration curve was linear over the concentration range ensured in the method validation studies with the correlation coefficient> 0.99. Accuracy of calibration and routine QC samples in each assay run met the acceptance criteria at all the laboratories. The % RE of calibration samples was within± 15% in all the methods. Cross validation QC samples were assayed at each laboratory with nominal concentration blinded and results are shown in Table 3. Accuracy of cross validation QC samples was within± 15% as recommended by European Medicines Agency [6] or US Food and Drug Administration [7] except the low concentration sample at the laboratory C (15.3%). However, the slight deviation is acceptable since regulators in Japan allow % RE within± 20% in cross validation studies [18].

**Table 3:** Percent relative error of cross validation quality control samples for lenvatinib assay.

**Laboratory**	**A**	**B**	**C**	**D**	**E**		
**Method**	**A**	**B**	**C**	**D**	**E1**	**E2**	**E3**
**Blank**	NA	NA	NA	ND	NA	NA	NA
**Low QC**	10.2	3.3	15.3	− 6.2	− 1.4	8.8	− 0.4
**Mid QC**	1.2	3.7	2.9	− 1.0	− 1.5	7.0	− 1.9
**High QC**	− 0.5	1.3	3.3	0.2	1.5	− 5.5	6.5

Data represent the mean relative error of cross validation quality control (QC) samples at each concentration (n = 3/concentration except laboratory D (n = 2)).

Concentration of cross validation QC samples were 0 (blank), 0.5 (low QC), 10 (mid QC), and 200 (high QC) ng/mL at the laboratories A, B, C, and E while 0.25 (low QC), 20 (middle QC), and 320 (high QC) ng/mL at the laboratory D.

NA: Not available due to lenvatinib concentrations below lower limit of quantification; ND: Not determined.

**Fig. 2:**
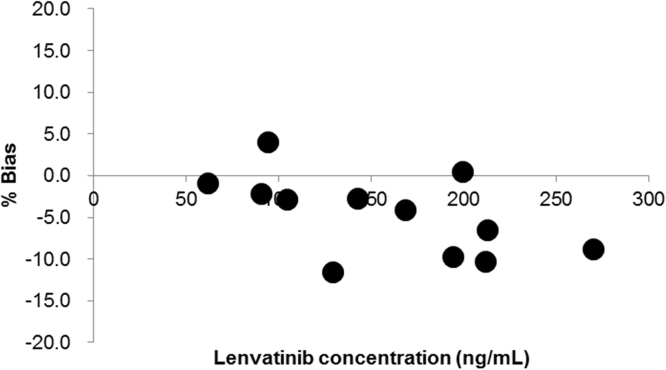
Percentage biases of lenvetinib concentrations between two laboratories in the assay of post-dose samples. Lenvatinib concentrations in 12 post-dose clinical plasma samples were determined at laboratories A and D. Mean plasma lenvatinib concentrations at the two laboratories and the corresponding percentage biases are plotted on the x and y-axes, respectively.

A total of 12 cross validation post-dose samples were assayed at the laboratories A and D. Lenvatinib concentrations ranged from 61.8 ng/mL to 258 ng/mL at the laboratory A while from 62.4 ng/mL to 282 ng/mL at the laboratory D (Fig. 2). Percentage biases between the two laboratories ranged from − 11.6 to 4.0%, which were well below the criteria (within± 20%).

## 4 Discussion

A bioanalytical method of lenvatinib in human plasma was first established at Eisai Co., Ltd (Tokyo, Japan) and was transferred to the laboratory A for the method validation study. After completion of the validation study at the laboratory A, the method was transferred to other laboratories for the method validation with some modifications if required (e.g. analytical column, mobile phase compositions, and gradient conditions, etc). Although there were some changes in the extraction and assay conditions, validation parameters at all the bioanalytical laboratories met the acceptance criteria, which ensured robustness of the assay method used in sample assay supporting clinical studies of lenvatinib.

In the carryover assessment in the methods E1 and E2, carryover peaks with> 20% LLOQ were detected in blank samples injected just after the ULOQ samples although reasons remain to be evaluated. As the dynamic ranges in the methods E1 (2000-fold) and E2 (1000-fold) were not wider than the other method in which no carryover was observed, the carryover is not ascribed to wider dynamic ranges. It is generally believed that carryover can be overcome by optimized injector washing solvents. Two types of injector washing solvents were used in the method E1; 1% trifluoroacetic acid in isopropanol-acetonitrile-water-methanol-acetone-dimethyl sulphoxide (1:1:1:1:0.5, v/v/v/v/v) and 0.05% formic acid in methanol-water (1:1, v/v). In the method E2, 0.1% formic acid in methanol followed by 0.1% formic acid in 50%methanol was used as an injector washing solvent. Although increased fraction of some organic solvent (e.g. isopropanol) might overcome carryover issue, unfortunately, these washing solvents did not address the carryover issue. In order to address the carryover issue in sample assay supporting clinical trials, potential impacts of carryover on lenvatinib concentrations in every sample were evaluated. The acceptance criterion was that the overestimated lenvatinib level due to carryover was less than 20% of the determined lenvatinib levels. When there were impacts on lenvatinib concentrations in samples by carryover, the samples in question were subjected to reanalysis.

A cross validation study is important when more than one laboratory is involved in sample assay during the course of clinical trials and when multiple assay methods are used in sample assay for a single study. Although a cross validation study is required by bioanalytical guidelines from the regulatory point of view [6], [7], pharmacokinetic data cannot be integrated or compared among multiple clinical trials unless bioanalytical data are comparable among the methods and laboratories. A number of cross validation studies have been reported in previous publications: typical cross validation studies included the comparison between the methods using different assay platforms (e.g. LC-MS/MS *vs* ELISA [11]), ones to compare methods using different detection equipment (*e.g.* LC-MS/MS *vs* LC with ultraviolet or fluorescence detection [10], [12], [13]), ones in the same platform with different analyte sets (*e.g*. single assay *vs* n-in-1 assay [16], [17]), one with different matrices (*e.g*. serum *vs* plasma [8]), and one using the same platforms with some modifications among laboratories [15]. The present study is categorized to the last type; a cross validation study using the same platform (*i.e*. LC-MS/MS) with some method modifications.

In the present study, a cross validation study was carried out using both QC samples and clinical post-dose samples as recommended by the guideline [7]. QC samples assayed at each laboratory were within the acceptance criteria. Although limited volume of post-dose samples would not allow post-dose samples to be assayed across all the laboratories, results in this study demonstrated that assay data were comparable between the two laboratories, including post-dose samples. Collectively, these findings in the cross validation study using both QC samples and post-dose samples suggest that plasma lenvatinib concentrations can be compared across the bioanalytical laboratories and clinical studies.

## 5 Conclusions

Results in the cross validation study were within the acceptance criteria, thus bioanalytical data of lenvatinib in human plasma can be compared across clinical studies.
